# Supplementary material for: Foliage of Tropical Trees and Shrubs and Their Secondary Metabolites Modify In Vitro Ruminal Fermentation, Methane and Gas Production without a Tight Correlation with the Microbiota
Source: Animals (Basel). 2022 Sep 30;12(19):2628. doi: 10.3390/ani12192628 (PMC9559637; doi:10.3390/ani12192628)
Supplement: Supplementary file 1 [file animals-12-02628-s001.zip › Supplementary Table S1. Primers.pdf]

Supplemental Table S1. List of primers used for the quantification of microbial populations by ddPCR

Primer name	Primer Sequence	Amplicon size	Target group	Reference
16S bacteria Fwd	5′GACTCCTACGGGAGGCAG3′	From 175 to 200 pb	α-bacteria<br>γ-bacteria<br>Firmicutes Bacteroidetes Actinobacteria *etc.*	Petrova *et al*. [1]
16S bacteria Rev	5′GTATTACCGCGGCTGCTG3′			
23S bacteria Fwd	5′GAGTAGGACGGAGCACGAG3′	161 pb	α-bacteria<br>γ-bacteria<br>Firmicutes Bacteroidetes Actinobacteria *etc.*	Petrova *et al*. [1]
23S bacteria Rev	5′CTTGTACGCATACGGTTTCAG3′			
16S archaea Fwd	5′AATTGGCGGGGGAGCAC3′	From 137 to 144 pb	Archaeas: Euryarchaeota and Crenarchaeota including: *Methanosphaera stadtmanae*, *Methanobrevibacter sp.*, *Methanosarcina sp.*	Bayer *et al*. [2]
16S archaea Rev	5′GGCCATGCACCWCCTCTC3′			
16S RO Fwd	5′GGGGCTAATACCGGATAGATGATT3′	459 bp	*Methanobrevibacter* RO group that includes: *M. ruminantium*, *M. olleyae*	Danielsson *et al*. [3]
16S RO Rev	5′CGACCTCAAGTTCAACAGTATCAC3′			
16S SGMT Fwd	5′TCCGTAGCCGGTTTAATAAGTCT3′	From 611 to 614 pb	*Methanobrevibacter* SGMT group that includes: *M. smithii*, *M. gottschalkii*, *M. millerae*, *M. thaueri*	Danielsson *et al*. [3]
16S SGMT Rev	5′TTCCTCCTATTTATCATAGGCGG3′			
mrcA Fwd	5′TTCGGTGGATCDCARAGRGC3′	160 bp	*methyl co-enzyme M reductase alpha subunit* (*mcrA*) gene	Denman *et al*. [4]
mrcA Rev	5′GBARGTCGWAWCCGTAGAATCC3′			
18S fungi Fwd	5′GAGGAAGTAAAAGTCGTAACAAGGTTTC3′	From 110 to 188 pb	Mainly anaerobic fungi including *Neocallimastix sp.*, *Orpinomyces sp.*, *Anaeromyces sp.*	Denman and McSweeney [5]
18S fungi Rev	5′CAAATTCACAAAGGGTAGGATGATT3′			
18S protozoa Fwd	5′CTTTCGATGGTAGTGTATTGGACTAC3′	From 1363 to 1459 bp	Several protozoa including *Epiphyllum sp.*	Karnati *et al*. [6]
18S protozoa Rev	5′TGATCCTTCTGCAGGTTCACCTAC3′			

References

1. Petrova, O. E.; Garcia-Alcalde, F.; Zampaloni, C.; Sauer, K. Comparative Evaluation of RRNA Depletion Procedures for the Improved Analysis of Bacterial Biofilm and Mixed Pathogen Culture Transcriptomes. *Sci Rep* **2017**, *7* (1), 41114. https://doi.org/10.1038/srep41114.
2. Bayer, K.; Kamke, J.; Hentschel, U. Quantification of Bacterial and Archaeal Symbionts in High and Low Microbial Abundance Sponges Using Real-Time PCR. *FEMS Microbiol Ecol* **2014**, *89* (3), 679–690. https://doi.org/10.1111/1574-6941.12369.
3. Danielsson, R.; Dicksved, J.; Sun, L.; Gonda, H.; Müller, B.; Schnürer, A.; Bertilsson, J. Methane Production in Dairy Cows Correlates with Rumen Methanogenic and Bacterial Community Structure. *Front. Microbiol.* **2017**, *8*. https://doi.org/10.3389/fmicb.2017.00226.
4. Denman, S. E.; Tomkins, N. W.; McSweeney, C. S. Quantitation and Diversity Analysis of Ruminal Methanogenic Populations in Response to the Antimethanogenic Compound Bromochloromethane: Monitoring of Rumen Methanogenic Archaea. *FEMS Microbiol Ecol* **2007**, *62* (3), 313–322. https://doi.org/10.1111/j.1574-6941.2007.00394.x.
5. Denman, S. E.; McSweeney, C. S. Development of a Real-Time PCR Assay for Monitoring Anaerobic Fungal and Cellulolytic Bacterial Populations within the Rumen: Real-Time PCR Assay of the Rumen Anaerobic Fungal Population. *FEMS Microbiol Ecol* **2006**, *58* (3), 572–582. https://doi.org/10.1111/j.1574-6941.2006.00190.x.
6. Karnati, S. K. R.; Yu, Z.; Sylvester, J. T.; Dehority, B. A.; Morrison, M.; Firkins, J. L. Technical Note: Specific PCR Amplification of Protozoal 18S RDNA Sequences from DNA Extracted from Ruminal Samples of Cows. *J Anim Sci* **2003**, *81* (3), 812–815. https://doi.org/10.2527/2003.813812x.
